# Comparison of Blood Profiles of γ-Oryzanol and Ferulic Acid in Rats after Oral Intake of γ-Oryzanol

**DOI:** 10.3390/nu11051174

**Published:** 2019-05-25

**Authors:** Takumi Kokumai, Junya Ito, Eri Kobayashi, Naoki Shimizu, Hiroyuki Hashimoto, Takahiro Eitsuka, Teruo Miyazawa, Kiyotaka Nakagawa

**Affiliations:** 1 Food and Biodynamic Chemistry Laboratory, Graduate School of Agricultural Science, Tohoku University, Sendai, Miyagi 980-8572, Japan; t.koku@dc.tohoku.ac.jp (T.K.); junyai@tohoku.ac.jp (J.I.); eri.k@dc.tohoku.ac.jp (E.K.); nshimizu@dc.tohoku.ac.jp (N.S.); takahiro.eiteuka.a1@dc.tohoku.ac.jp (T.E.); 2 Tsuno Food Industrial Co., Ltd., Ito-Gun, Wakayama 649-7194, Japan; hirohas@tsuno.co.jp; 3 Food and Biotechnology Innovation Project, New Industry Creation Hatchery Center (NICHe), Tohoku University, Sendai, Miyagi 980-8579, Japan; miyazawa@m.tohoku.ac.jp; 4 Food and Health Science Research Unit, Graduate School of Agricultural Science, Tohoku University, Sendai, Miyagi 980-0845, Japan

**Keywords:** γ-oryzanol, ferulic acid, absorption and metabolism, HPLC-MS/MS, rice bran

## Abstract

γ-Oryzanol (OZ), a bioactive phytochemical abundant in cereals such as rice, has been reported to be mainly hydrolyzed to ferulic acid (FA) in the body. Meanwhile, in our previous study, we revealed that a part of OZ is absorbed into the body and exists in its intact form. However, the comprehensive absorption profile of OZ and its metabolites (e.g., FA) after OZ intake has not been fully elucidated yet. Therefore, in this study, we measured the concentrations of OZ, FA, and FA conjugates (i.e., FA sulfate and glucuronide) in the blood of rats with the use of HPLC-MS/MS after a single oral administration of 300 µmol/kg body weight of rice bran OZ (RBOZ). As a result, intact OZ along with FA and FA conjugates existed in the blood, which implied that these constituents may all contribute to the physiological effects under OZ intake. Additionally, when an equimolar amount of FA (300 µmol/kg body weight) was administered, it was found that the absorption profile of FA was significantly different from that when RBOZ was administered.

## 1. Introduction

γ-Oryzanol (OZ), a mixture of ferulic acid (FA) esters of triterpene alcohols and plant sterols, is characteristically contained in cereals such as rice. Since OZ was discovered from rice oil in 1954 [1], about ten kinds of OZ molecular species have been reported to date [2,3]. In particular, cycloartenyl ferulate (CA-FA), 24-methylenecycloartanyl ferulate (24MCA-FA), campesteryl ferulate (Camp-FA), and β-sitosteryl ferulate (Sito-FA) are the major components of rice OZ [4,5,6]. Numerous studies have reported the physiological effects of OZ, including anti-oxidative [7], anti-diabetic [8], anti-carcinogenic [9], neuroprotective [10], and lipid lowering effects [11]. Furthermore, in Japan, OZ has been used as a medicinal drug for hyperlipidemia and psychosomatic diseases.

In order to further understand the physiological effects of OZ, it is important to reveal the abundance of OZ and its metabolites in the blood and target tissues in human and animals. To investigate the blood OZ profile, Fujiwara et al. orally administered ^14^C-labeled OZ and measured its radioactivity in rabbits [12]. The result showed that the radioactivity in blood was induced mainly by FA, thus implying the hydrolysis of OZ to FA in the body (i.e., during intestinal absorption or blood circulation). Meanwhile, using high-performance liquid chromatography-tandem mass spectrometry (HPLC-MS/MS), we were able to determine the OZ level in biological samples such as plasma. Using this method, we revealed that a part of OZ is absorbed into the body and exists in the blood (at nM order) [1] and organs in the intact form in mice [13]. Taking these forms of evidence into consideration, it was hypothesized that a part of OZ avoids the hydrolysis to FA and remains in its intact form in blood and organs.

**Figure 1:**
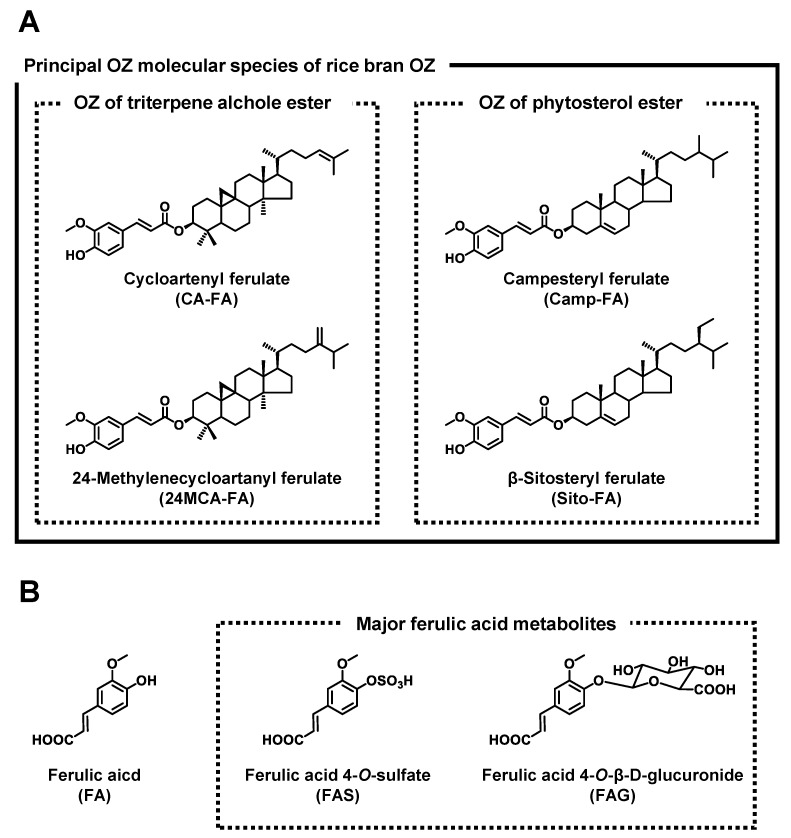
Chemical structures of principal γ-oryzanol (OZ) molecular species in rice bran (cycloartenyl ferulate, 24-methylenecycloartanyl ferulate, campesteryl ferulate, and β-sitosteryl ferulate) (**A**). Chemical structures of ferulic acid and major ferulic acid conjugates (ferulic acid sulfate and glucuronide) (**B**).

To further prove this hypothesis, it is necessary to determine the profiles of OZ together with FA in biological samples after OZ intake. To the best of our knowledge, such information has not yet been available, except for our previous study in which we measured the plasma concentrations of OZ and FA at a single time point after administration of OZ [13]. While it was interestingly implied that the plasma concentration of OZ and FA were similar, comprehensive insight into the absorption patterns of OZ and FA over a period of time was not available. Moreover, FA is known to be metabolized into a number of metabolites in the body, with FA sulfate and glucuronide as the major FA conjugates (Figure 1) [14,15]. Therefore, in the present study, we aimed to determine the blood profile of OZ, FA, and FA conjugates (FA sulfate and glucuronide) at multiple time points after rice bran OZ (RBOZ) intake using HPLC-MS/MS. Additionally, the blood profile after administration of an equimolar amount of FA was analyzed to compare the absorption profile of OZ with FA. The results of this study may help predict which active molecules (intact OZ, FA, etc.) play a major role in the physiological effects of OZ.

## 2. Materials and Methods 

### 2.1. Materials

RBOZ was received from Tsuno Food Industry Co., Ltd. (Wakayama, Japan). The purity of RBOZ as OZ equivalent was determined as 99.7% based on ultraviolet (UV) spectrophotometric (325 nm) analysis [16]. Standard CA-FA was purchased from Wako Pure Chemical Industries, Ltd. (Osaka, Japan). Other OZ standards (i.e., 24MCA-FA, Camp-FA, and Sito-FA) were obtained in accordance with our previous report [13]. FA and FA 4-*O*-β-d-glucuronide disodium salt were obtained from Toronto Research Chemicals, Inc. (Toronto, ON, Canada), and FA 4-*O*-sulfate disodium salt was purchased from Carbosynth, Ltd. (Berkshire, U.K.). Soybean oil was purchased from Wako Pure Chemical Industries, Ltd. All other reagents were of the highest grade available.

### 2.2. Animal Study

Male Sprague–Dawley rats (11 weeks old; CLEA Japan, Tokyo, Japan) weighing 370–390 g were housed with free access to commercial chow diet (AIN93G, Research Diets, Inc., New Brunswick, NJ, USA) and distilled water in a room with controlled temperature (20–25 °C) and lighting (lights on from 08:00 to 20:00). After 1 week of acclimatization, rats were fasted for 12 h and divided into two groups: the first group was administered with RBOZ dispersed in soybean oil (32.8 g/kg) via gastric intubation (300 µmol/kg body weight) (RBOZ group, *n* = 6) and the second group was given an oral dose of FA dispersed in soybean oil (16.4 g/kg) via gastric intubation (300 µmol/kg body weight) (FA group, *n* = 6). At 0, 0.5, 1, 3, 6, 9, and 12 h after sample administration, 440 µL of blood was collected via tail vein by capillary tubes. During the test period, all animals were given free access to distilled water. Plasma was isolated from the collected blood by centrifugation at 1000× *g* for 20 min at 4 °C and stored at −80 °C until use. This study was conducted according to protocols approved by the Institutional Committee for the Tohoku University Ethics Review Board (2018AgA-040).

### 2.3. Extraction of Plasma OZ, FA, and FA Conjugates

Extraction of OZ was carried out based on our previous method [1,13] with slight modifications. Plasma (40–60 µL) was added with 0.9% KCl aqueous solution to reach a total volume of 600 µL. Then, 2.4 mL of chloroform–methanol (2:1, *v*/*v*) containing 0.002% butylated hydroxytoluene was added to the diluted plasma. The mixture was subjected to centrifugation at 2000× *g* for 20 min at 4 °C resulting in the formation of two layers. The lower chloroform layer (lipid fraction) was collected, while the remaining upper aqueous layer was re-extracted using 1.4 mL of chloroform–methanol (10:1, *v*/*v*). The mixture was then subjected to centrifugation at 2000× *g* for 20 min at 4 °C to obtain two layers. The lower layer was collected and combined with the previously collected lower layer from the first extraction. The obtained total lipid extract was evaporated under nitrogen gas and re-dissolved in 1 mL of hexane–chloroform (9:1, *v*/*v*). To further purify the extract, 900 µL of the total lipid solution was loaded onto a Strata SI-1 Silica cartridge (Phenomenex Inc., Torrance, CA, USA) equilibrated with 1.5 mL of hexane–chloroform (9:1, *v*/*v*). The cartridge was rinsed with 1.5 mL hexane–chloroform (9:1, *v*/*v*) and OZ was eluted with 1.5 mL of hexane–2-propanol (7:3, *v*/*v*). The eluent was evaporated under nitrogen gas, and the residue was dissolved in 200 µL of methanol. The solution was cooled on ice for 15 min and was filtered by Nanosep with Bio-Inert membrane (0.2 µm, Pall Corporation, NY, USA). Finally, a 20-µL aliquot of the OZ fraction was subjected to HPLC-MS/MS analysis.

For the extraction of FA and FA conjugates, plasma (40–80 µL) was added with 400 μL of 1% formic acid methanol solution, vortexed, and subjected to centrifugation at 13,000× *g* for 10 min at 4 °C. The upper layer was collected, while 300 μL of 1% formic acid methanol solution was added to the remaining residue, vortexed, and subjected to centrifugation at 13,000× *g* for 10 min at 4 °C. The upper layer was collected and combined with the previously collected upper layer from the first extraction. Chloroform (200 µL) was added to the obtained methanol extract, evaporated under nitrogen gas, and re-dissolved in 1 mL of 1% formic acid methanol–water (1:9, *v*/*v*). To further purify the extract, 900 µL of the mixture was loaded onto a Strata-X column (Phenomenex Inc., Torrance, CA, USA) conditioned with 1% formic acid methanol solution and equilibrated with 1% formic acid aqueous solution. After loading the sample, the cartridge was rinsed with 1.5 mL of 1% formic acid aqueous solution followed by elution of FA and FA conjugates with 1.5 mL methanol–water (9:1, *v*/*v*). Chloroform (4 mL) was added to the eluent, evaporated, and the residue was dissolved in 300 µL of acetonitrile–water (1:9, *v*/*v*). Finally, a 20-µL aliquot of this solution was subjected to HPLC-MS/MS analysis.

### 2.4. HPLC-MS/MS Analysis

The HPLC-MS/MS system consisted of a Shimadzu liquid chromatography system (Shimadzu, Kyoto, Japan) and a 4000 QTRAP mass spectrometer (SCIEX, Tokyo, Japan). MS/MS parameters were optimized under negative ion electrospray ionization (see Table S1 in the Supplementary Materials section).

OZ molecular species were separated using a HPLC column (COSMOSIL 2.5C18-MS-II, 2.5 µm, 2.0 ID × 100 mm; Nacalai Tesque, Inc., Kyoto, Japan) at 40 °C with a flow rate of 0.5 mL/min. Gradient elution was performed using two mobile phases: A, methanol containing 1% acetic acid; and B, 2-propanol. The gradient solvent system was as follows: 0–5 min, 0% B; 5.01–7.00 min, 100% B; 7.01–10.00 min, 0% B. The standard curves of OZ molecular species were prepared in the range of 1 to 100 fmol.

FA and FA conjugates were separated using a HPLC column (InertSustain C8, 2 µm, 2.1 ID × 100 mm; GL Science, Tokyo, Japan) at 40 °C with a flow rate of 0.3 mL/min. Gradient elution was performed using two mobile phases: A, water containing 0.1% formic acid; and B, acetonitrile containing 0.1% formic acid. The gradient solvent system was as follows: 0–7 min, 10–25% B linear; 7.01–9.50 min, 100% B; 9.51–12.00 min, 10% B. Total flow was set as follows: 0–7 min, 0.3–0.4 mL/min linear; 7.01–9.50 min, 0.4 mL/min; 9.51–12.00 min, 0.3 mL/min. The standard curves of FA and FA conjugates were prepared in the range of 0.01 to 60 pmol.

### 2.5. Statistics

Data are expressed as means ± standard errors (SE). Data regarding the concentration of plasma OZ molecular species and FA and FA conjugates were analyzed by one-way ANOVA followed by Tukey’s test. Differences were considered significant at *p* < 0.05.

## 3. Results and Discussion

### 3.1. Analysis of RBOZ by HPLC-MS/MS

OZ is a mixture of FA esters of triterpene alcohols and plant sterols. Since OZ was discovered from rice oil in 1954 [1], numerous studies have reported the beneficial properties of OZ, including its anti-oxidative effect [7]. To further understand the beneficial properties of OZ, it is important to reveal the abundance of OZ and its metabolites in the body. Considering our previous reports [1,13] as well as others [12] about the absorption and metabolism of OZ, it was hypothesized that a part of OZ avoids hydrolysis to FA and remains in blood and organs in its intact form. To further prove this hypothesis, it is necessary to determine the profiles of OZ together with FA in biological samples after OZ intake. Therefore, in this study, we aimed to determine the blood profile of OZ, FA, and FA conjugates at multiple time points.

Prior to administration, the molar ratio of OZ molecular species in RBOZ was determined to administer OZ accurately at a dose of 300 µmol/kg body weight. Previous studies have described CA-FA, 24MCA-FA, Camp-FA, and Sito-FA as the major OZ species in rice bran [4,5,6], and hence, we analyzed these constituents in RBOZ using HPLC-MS/MS. As a result, the molar ratios of CA-FA, 24MCA-FA, Camp-FA, and Sito-FA in RBOZ were 44.3%, 40.4%, 9.4%, and 6.0%, respectively. Based on these values, 300 µmol/kg body weight (i.e., CA-FA, 132.9 µmol; 24MCA-FA, 121.2 µmol; Camp-FA, 28.2 µmol; and Sito-FA, 18 µmol) of RBOZ accounted for 182 mg/kg body weight (i.e., CA-FA, 80.1 mg; 24MCA-FA, 75.1 mg; Camp-FA, 16.2 mg; and Sito-FA, 10.6 mg). This dosage (182 mg/kg body weight) was comparable to various previous OZ single oral administration tests that were conducted in the dosage ranging between 25–1600 mg/kg body weight [17,18,19,20], and was a dosage that may exert physiological effects considering that previous studies administering OZ at 100 mg/kg body weight/day resulted in lipid-lowering effects [21].

### 3.2. Plasma Profiles of OZ after Administration of RBOZ

**Figure 2:**
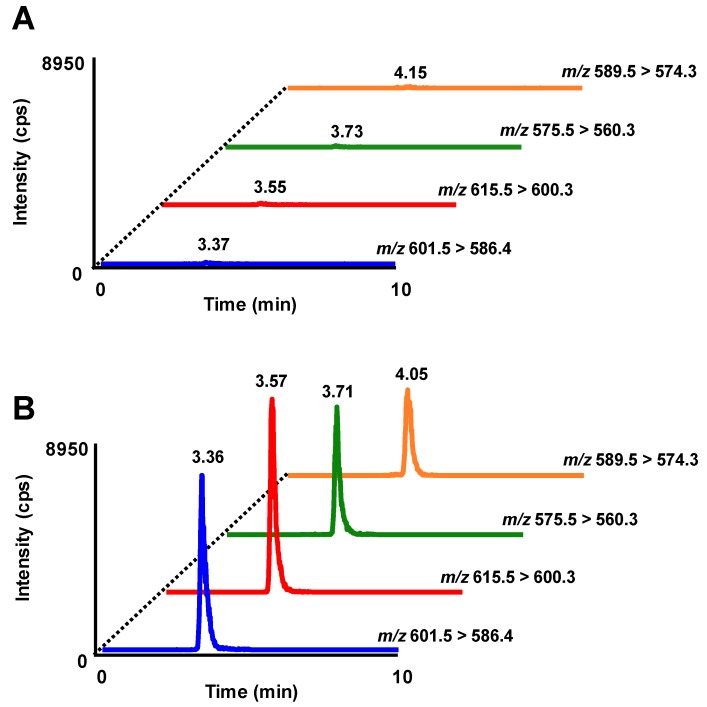
Typical multiple reaction monitoring (MRM) chromatograms analyzing OZ molecular species in plasma before (**A**) and 9 h after rice bran OZ (RBOZ) administration (**B**). Each MRM pair represents analysis of the following OZ molecular species: *m*/*z* 601.5 > 586.4, cycloartenyl ferulate; *m*/*z* 615.5 > 600.3, 24-methylenecycloartanyl ferulate; *m*/*z* 575.5 > 560.3, campesteryl ferulate; *m*/*z* 589.5 > 574.3, β-sitosteryl ferulate.

**Figure 3:**
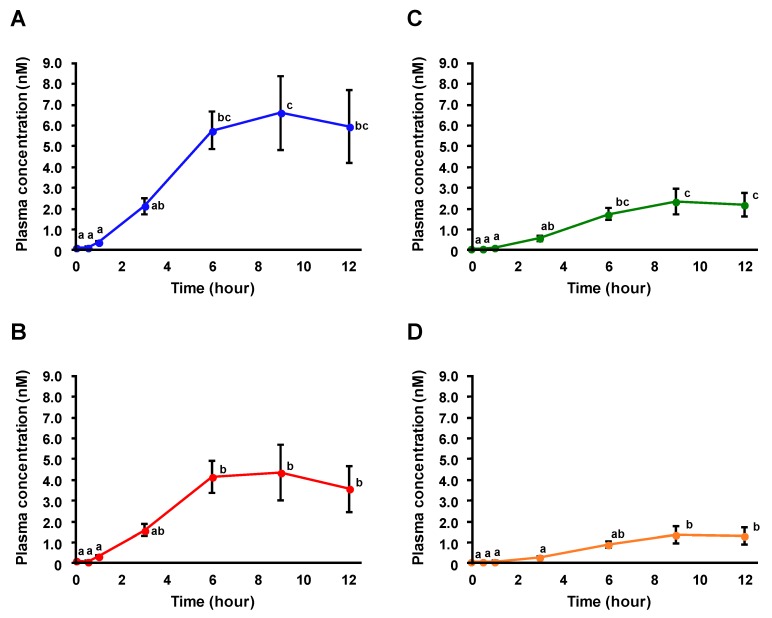
Time-dependent changes in plasma concentrations (nM) of each OZ molecular species (CA-FA (**A**), 24MCA-FA (**B**), Camp-FA (**C**), and Sito-FA (**D**)) after RBOZ administration. Data are shown as means ± SE (*n* = 6) and different letters represent significant difference at *p* < 0.05.

RBOZ (300 µmol/kg body weight) was administered to male Sprague–Dawley rats (12 weeks old, *n* = 6), and at first, the absorption profile of OZ in rat plasma was investigated using HPLC-MS/MS. The detection limit of each OZ molecular species was 1 fmol. Typical multiple reaction monitoring (MRM) chromatograms of OZ molecular species in plasma before and 9 h after RBOZ administration are shown in Figure 2. While peaks were hardly detected before the administration, all OZ molecular species were found at higher intensities 9 h after RBOZ administration. Hence, taken together with our previous study, it was confirmed that OZ was absorbed into blood in the intact form. Plasma concentrations of OZ molecular species were determined based on external calibration curves of each standard. Time-dependent changes in plasma concentrations of each OZ molecular species are demonstrated in Figure 3. To the best of our knowledge, this is the first study showing time-dependent concentration changes of intact OZ molecular species after a single dose administration of OZ. Concentrations of each OZ molecular species in plasma were found to gradually increase until 9 h, when OZ concentrations reached a maximum. While studies regarding the absorption and metabolism of OZ are limited, the absorption and metabolism of sterols (especially plant sterols whose structures are similar with that of OZ) have been investigated. It has been reported that the lymphatic transfer rate of β-sitosterol significantly increased until after 9 h of administration [22], suggesting that a similar trend can also be observed in plasma concentrations of β-sitosterol. Comparing such results with the current study investigating the absorption profile of OZ, it was implied that the absorption speeds of OZ and plant sterols are similar.

The area under the plasma concentration-time curve (AUC) for each OZ molecular species is shown in Table 1. The relative proportion of OZ molecular species in plasma was calculated with the formula below:

(1) $$\mathrm{Relative}\;\mathrm{proportion}\;\left(\%\right)\;=\;\frac{\mathrm{AUC}\;\mathrm{of}\;\mathrm{OZ}\;\mathrm{molecular}\;\mathrm{species}}{\mathrm{Sum}\;\mathrm{of}\;\mathrm{AUC}\;\mathrm{of}\;\mathrm{all}\;\mathrm{OZ}\;\mathrm{molecular}\;\mathrm{species}}\;\times \;100$$

Interestingly, it was found that the relative proportion of OZ molecular species in plasma was different to that of RBOZ (e.g., the relative proportion of 24MCA-FA and Camp-FA in plasma was 9.5% lower and 5.6% higher, respectively, compared to that of RBOZ). A similar observation was made in a previous study where different plant sterols were administered to rats; the lymphatic transfer rate of plant sterols differed between plant sterol molecular species [23]. Hence, it can be assumed that the difference in the relative proportion of OZ molecular species in plasma may be due to the different sterol/triterpene alcohol structure of OZ molecular species. This observation may be related to the aforementioned trend where the absorption speed of OZ was similar to that of sterols. Further studies are necessary to confirm whether the difference in absorption of OZ molecular species is induced by differences in structures.

### 3.3. Plasma Profiles of FA after Administration of RBOZ

**Figure 4:**
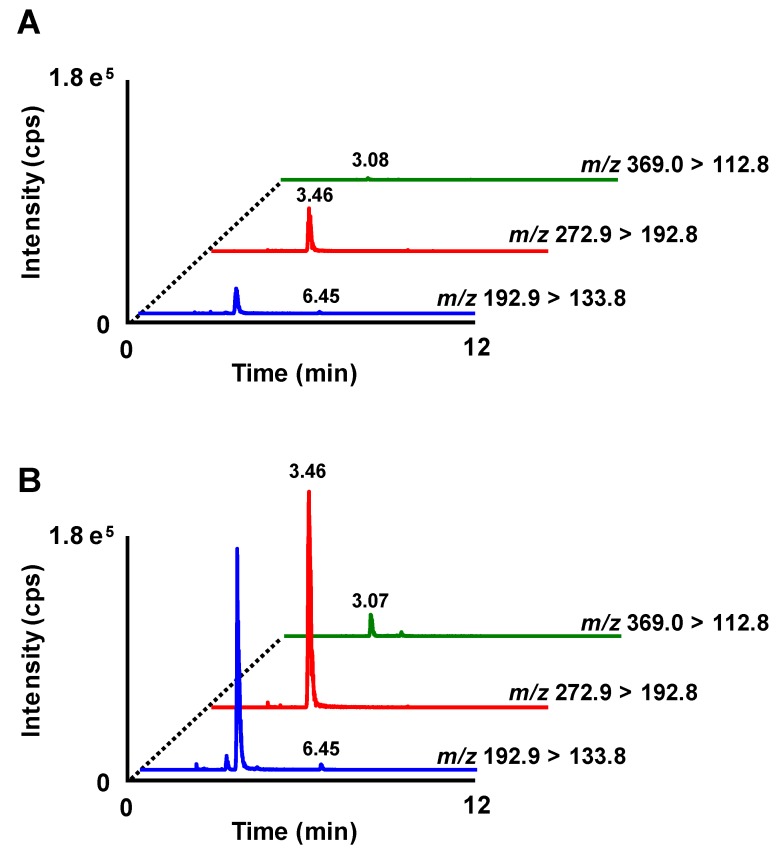
Typical MRM chromatograms of FA and FA conjugates in plasma before (**A**) and 6 h after RBOZ administration (**B**). Each MRM pair represents analysis of the following FA and FA conjugates: *m*/*z* 192.9 > 133.8, ferulic acid; *m*/*z* 272.9 > 192.8, ferulic acid 4-*O*-sulfate; *m*/*z* 369.0 > 112.8, ferulic acid 4-*O*-β-d-glucuronide.

**Figure 5:**
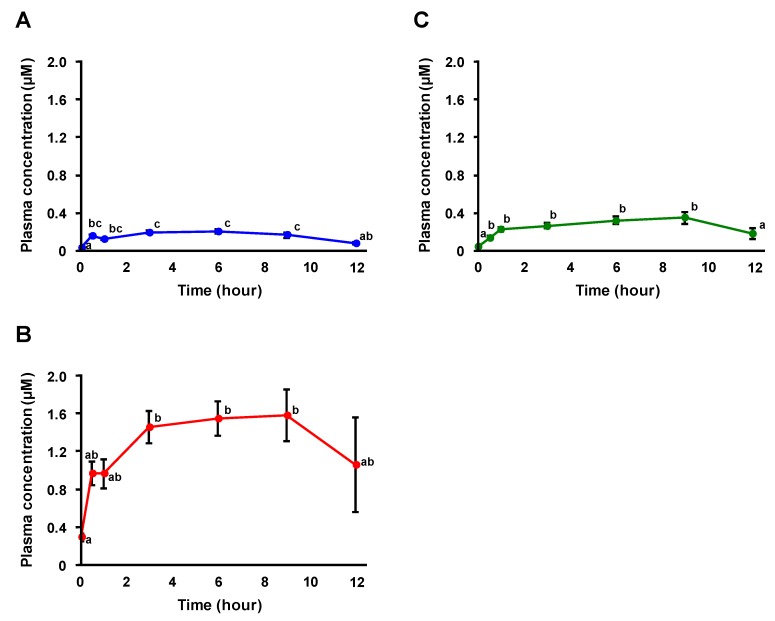
Time-dependent changes in plasma concentrations (μM) of FA and FA conjugates (FA (**A**), FAS (**B**), and FAG (**C**)) after RBOZ administration. Data are shown as means ± SE (*n* = 6) and different letters represent significant difference at *p* < 0.05.

The absorption profiles of FA and FA conjugates (i.e., FA 4-*O*-sulfate (FAS) and FA 4-*O*-β-d-glucuronide (FAG)) after RBOZ administration were also investigated using HPLC-MS/MS. The detection limits of FA and FA conjugates were as follows: FA (0.1 pmol), FAS (0.01 pmol), and FAG (0.05 pmol). Typical MRM chromatograms of FA and its conjugates in plasma before and 6 h after RBOZ administration are shown in Figure 4. While the peaks of FA, FAS, and FAG were hardly detected from plasma before administration, in the plasma 6 h after RBOZ administration, FAS and FAG were found at higher intensities along with a small peak of FA. Thus, it was found that OZ-derived FA was further metabolized to FA conjugates. To the best of our knowledge, this is the first evidence demonstrating that FA conjugates such as FAS and FAG appeared in the bloodstream after OZ intake. Time-dependent changes in concentrations of FA and its conjugates in plasma after RBOZ administration are shown in Figure 5. With regard to free FA, since the plasma concentrations were relatively low, only a slight increase over time was observed. Meanwhile, the plasma concentrations of FAS and FAG rapidly increased within approximately 1–3 h and stayed at a considerable level for a relatively long period (~12 h). It is important to note that the absorption profiles of FAS and FAG were similar to that of OZ in the time range of 3–12 h.

The AUCs of OZ, FA, and FA conjugates in plasma after RBOZ administration are shown in Table 2. FAS demonstrated the highest AUC, followed by FAG, FA, and OZ. It was found that the AUCs of FA conjugates were significantly higher than that of FA and OZ, which implied that OZ was hydrolyzed to FA but mainly existed in the form of FA conjugates, especially FAS and FAG, in the blood. Comparing the AUCs of FAS and FAG, FAS demonstrated a higher value, suggesting that FA is relatively susceptible to sulfate conjugation. With regard to the physiological effects of FA and its conjugates, it has previously been described in an in vitro assay that the anti-oxidative properties of FAS and FAG are significantly lower than that of free FA [24], and thus it has been generally considered that the conjugation of FA is an obstacle for FA to exert its functions. However, it has also been reported that FAS and FAG demonstrated larger physiological effects than free FA when their vasorelaxant and anti-oxidative effects in low density lipoprotein-cholesterol were examined [25,26]. Considering that the AUCs of FA conjugates were much higher than that of free FA and OZ, it is possible that FA conjugates also contribute in part to the physiological effects of OZ.

Comparing the absorption profiles of both OZ and FA after RBOZ administration, it was found that while OZ was hydrolyzed to FA and mainly existed as FA conjugates, a part of OZ remained in plasma until 12 h after RBOZ administration at nM order, which proved the hypothesis mentioned above that a part of OZ avoids the hydrolysis to FA and remains in its intact form inside the body of rats. While such results suggested that FA conjugates contribute in part to the physiological effects of OZ, since it was also found that a part of OZ avoids the hydrolysis and remains in blood, OZ in its intact form may also contribute to the physiological effects of OZ.

### 3.4. Plasma Profiles after Administration of FA

**Figure 6:**
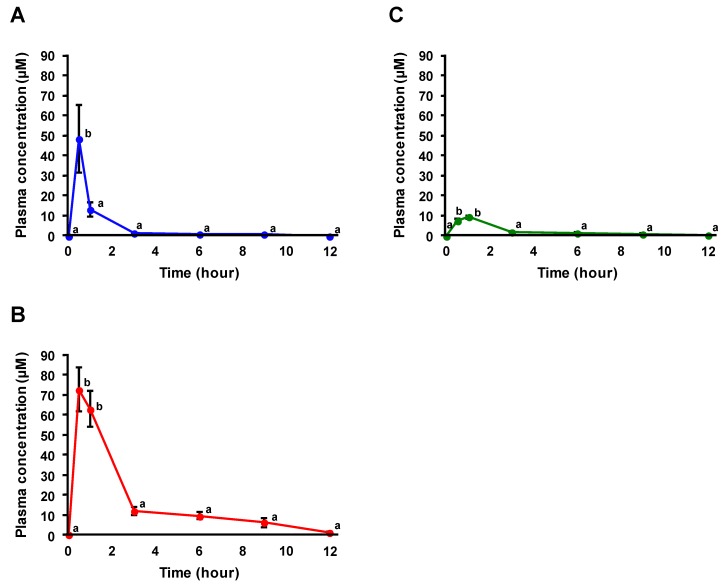
Time-dependent changes in plasma concentrations (μM) of FA and FA conjugates (FA (**A**), FAS (**B**), and FAG (**C**)) after FA administration. Data are shown as means ± SE (*n* = 6) and different letters represent significant difference at *p* < 0.05.

Previous studies have demonstrated that the administration of equal amounts of OZ and FA results in similar strengths of physiological effects [11,27,28]. Accordingly, an equimolar amount of FA was administered to compare the plasma profiles between the administration of RBOZ and FA. As shown in Figure 6, plasma concentrations of FA and its conjugates rapidly increased within 0.5 h of administration, as maximum concentrations (C_max_) of FA and its conjugates were typically achieved at 0.5 h. This rapid increase in concentrations of FA and its conjugates reflects the initial increase (within 1 h) in FA concentrations under RBOZ administration (Figure 5). Considering that the absorption of OZ was slower than that of FA (plasma OZ concentrations gradually increased until 9 h of administration; Figure 3), the initial increase in FA concentrations under RBOZ administration may suggest the hydrolysis of OZ to FA in the intestine before absorption into the body. On the other hand, whereas the concentration of FA and its conjugates under FA administration rapidly decreased after 0.5 h (Figure 6), that of FA and its conjugates under RBOZ administration stayed at a considerable level for a relatively long period (~12 h) (Figure 5). Since this absorption profile was similar to that of OZ under RBOZ administration (Figure 6), it can be suggested that a part of OZ was absorbed in its intact form into blood circulation where OZ was hydrolyzed into FA and its conjugates.

The AUCs of FA, FAS, and FAG after FA administration were found to be 30.2 times, 14.6 times, and 11.0 times higher compared to the RBOZ group, respectively (Table 3), demonstrating that the concentration of FA in blood under RBOZ administration was much lower than when FA was administered. This may be because the administered RBOZ is not effectively hydrolyzed to FA. On the other hand, in a previous study where rats were fed a diet supplemented with either FA or OZ (at equimolar concentrations), FA and OZ exhibited comparable effects against obesity, hyperlipidemia, hyperglycemia, and insulin resistance induced by high-fat and high-fructose diet intake. However, only OZ significantly decreased liver parameter and hepatic lipid contents, lowered serum levels of C-reactive protein and interleukin-6, and increased serum concentration of adiponectin [28]. Considering such past reports along with the findings of the current study where plasma concentrations of FA and FA conjugates were markedly lower after OZ intake compared to FA intake, it can be suggested that not only FA and FA conjugates but also OZ itself may contribute to the physiological effects under OZ intake.

## 4. Conclusions

In this study, the blood profiles of OZ, FA, and FA conjugates (FAS and FAG) after a single dose administration of RBOZ were first determined at multiple time points using HPLC-MS/MS. As a result, not only FA and its conjugates but also OZ was found from plasma, suggesting that a part of OZ avoids hydrolysis to FA and remains in blood. Additionally, the blood profile after administration of an equimolar amount of FA was analyzed to compare the absorption profile of OZ with FA. The absorption profile of FA was significantly different from that when RBOZ was administered, providing insights into how OZ is metabolized to FA and its conjugates in vivo. The results of this study may help predict which active molecules (intact OZ, FA, etc.) contribute to a major role in the physiological effects of OZ.

## Figures and Tables

**Table 1:** The area under the plasma concentration-time curve (AUC; expressed as nmol·h/L) and relative to the proportion of OZ molecular species in plasma after RBOZ administration.

Data	CA-FA	24MCA-FA	Camp-FA	Sito-FA
AUC (nmol·h/L)	51.5 ± 10.0	34.8 ± 7.6	16.9 ± 3.4	9.5 ± 2.1
Relative proportion (%)	45.7 ± 1.4	30.9 ± 0.8	15.0 ± 0.7	8.4 ± 0.3

Mean ± SE (*n* = 6).

**Table 2:** The AUC (expressed as µmol·h/L) of OZ, FA, and FA conjugates after RBOZ administration.

Data	Total OZ Species	FA	FAS	FAG
AUC (µmol·h/L)	0.11 ± 0.02	1.62 ± 0.12	13.58 ± 1.49	2.64 ± 0.35

Mean ± SE (*n* = 6).

**Table 3:** The AUCs (expressed as µmol·h/L) of FA, and FA conjugates after FA administration.

Data	FA	FAS	FAG
AUC (µmol·h/L)	48.9 ± 6.4	198.0 ± 13.6	29.0 ± 1.9

Mean ± SE (*n* = 6).

## Supplementary Materials

The following are available online at https://www.mdpi.com/2072-6643/11/5/1174/s1, Table S1: The analytical condition for HPLC-MS/MS.
