# Supplementary material for: Comparison of Blood Profiles of γ-Oryzanol and Ferulic Acid in Rats after Oral Intake of γ-Oryzanol
Source: Nutrients. 2019 May 25;11(5):1174. doi: 10.3390/nu11051174 (PMC6567117; doi:10.3390/nu11051174)
Supplement: Supplementary file 1 [file nutrients-11-01174-s001.pdf]

Table S1. The analytical condition for HPLC-MS/MS.

	CA-FA	24MCA-FA	Camp-FA	Sito-FA	FA	FAS	FAG
Precursor ion (m/z) [M-H]-	601.5	615.5	575.5	589.5	192.9	272.9	369.0
Product ion (m/z)	586.3	600.3	560.3	574.3	133.8	192.8	112.8
Source				ESI			
Ion polarity				Negative			
Declustering potential (V) *	−145	−145	−175	−155	−90	−77	−77
Entrance potential (V)	−10	−10	−10	−10	−10	−10	−10
Collision energy (V) *	−56	−58	−50	−52	−23	−21	−21
Collision cell exit potential (V) *	−27	−29	−23	−31	−4	−7	−3
Curtain gas (psi)	20	20	20	20	30	30	30
Collision gas (psi)	5	5	5	5	8	8	8
Ion spray voltage (V)	−4500	−4500	−4500	−4500	−4500	−4500	−4500
Temperature (°C)	700	700	700	700	700	700	700
Ion source gas 1 (psi)	50	50	50	50	60	60	60
Ion source gas 2 (psi)	80	80	80	80	80	80	80

ESI, electrospray ionization; CA-FA, cycloartenyl ferulate; 24MCA-FA, 24-methylencycloartanyl ferulate; Camp-FA, Campesteryl ferulate, Sito-FA, β-sitosteryl ferulate; FA, ferulic acid; FAS, ferulic acid 4-*O*-sulfate; FAG, ferulic acid 4-O-β-D-glucuronide.

Corresponding author

Kiyotaka Nakagawa

E-mail: nkgw@m.tohoku.ac.jp; Tel.: +81-22-757-4416
